# The Paddington Infirmary System

**Published:** 1915-02-20

**Authors:** A. Smith

**Affiliations:** 20 Gordon Place, High Street, Kensington.


### The Paddington Infirmary System.

*To the Editor of* The Hospital.

Sir,—Seeing Dr. Stewart's letter of Paddington Infirmary, I think his system very good of keeping records and notes of patients. I beg to state that I was trained in a large London infirmary, and have also been sister and midwife in two others, and find that they still adhere to the old bed-cards and do not get past notes.

In one case I was given a case-book and asked by the medical superintendent to take notes of all accident cases. But in my training school we had the old notes when the patient came in a second time. I think myself that they are very brief, and also that in some infirmaries the lack of surgical training is greatly to be lamented.—Yours faithfully, A. Smith.

20 Gordon Place, High Street, Kensington.
February 14.

